# Supplementary figures and images for: Molecular basis of neurodegeneration in a mouse model of Polr3-related disease
Source: eLife. 2024 Nov 5;13:RP95314. doi: 10.7554/eLife.95314 (PMC11537486; doi:10.7554/eLife.95314)

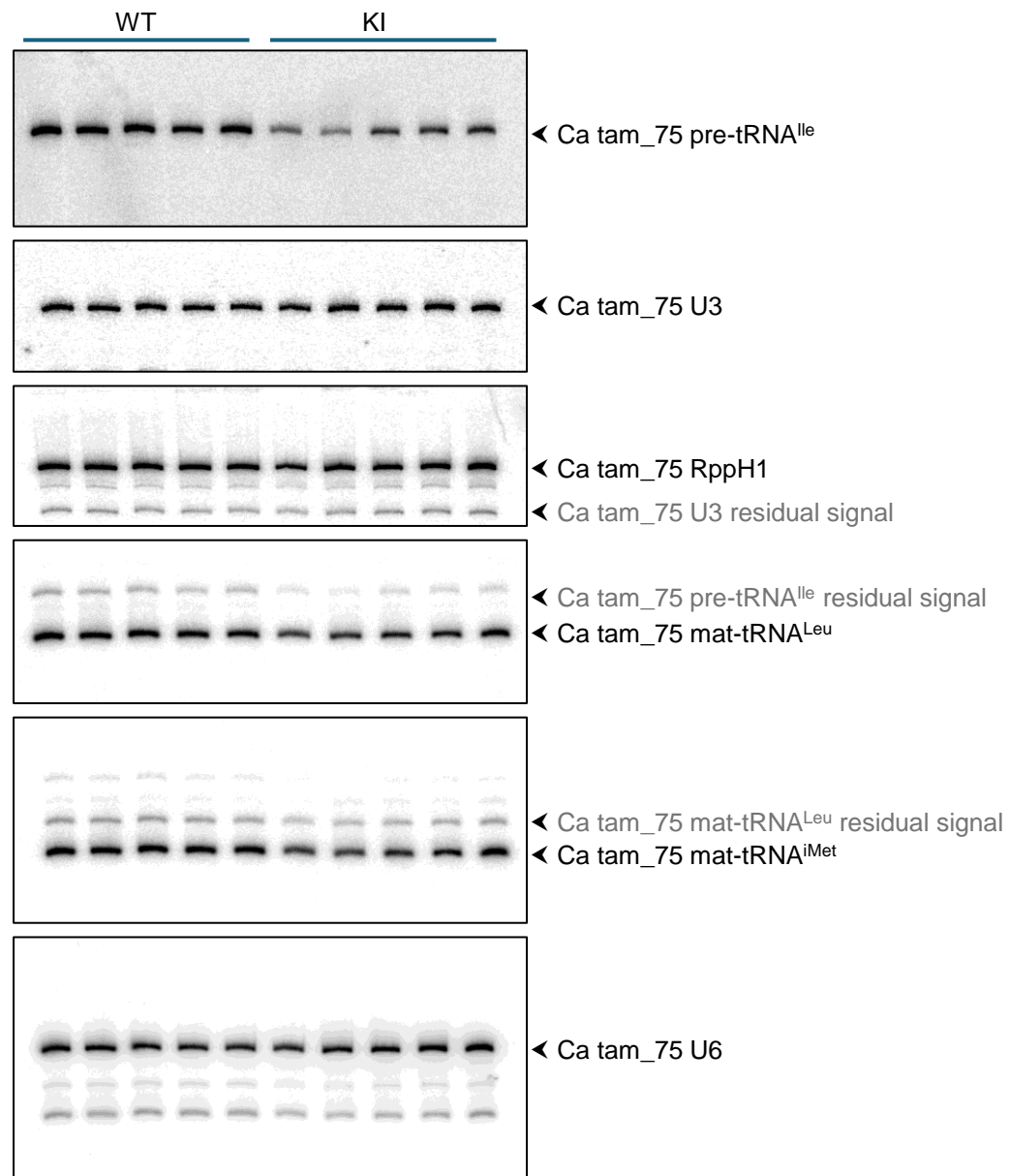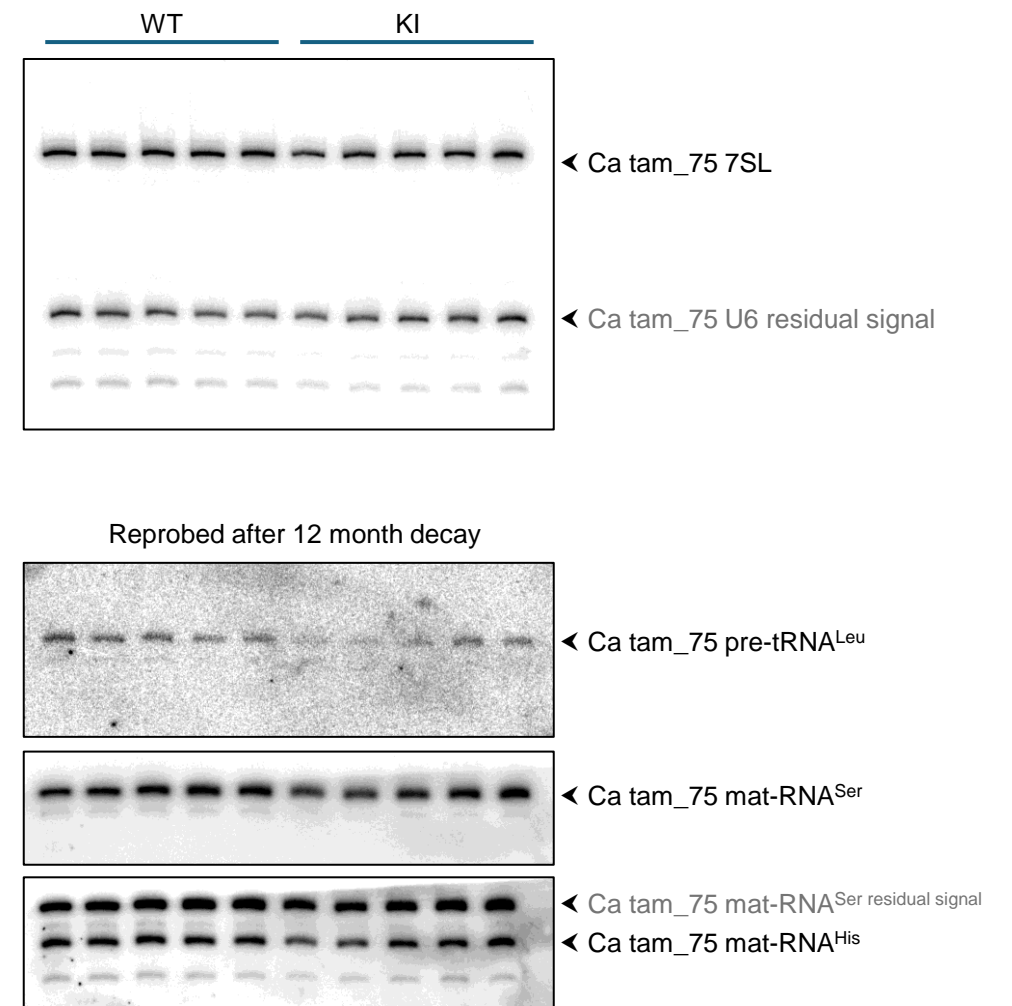

Supplement: Figure 3—source data 1. [file elife-95314-fig3-data1.pdf]

Cerebellum

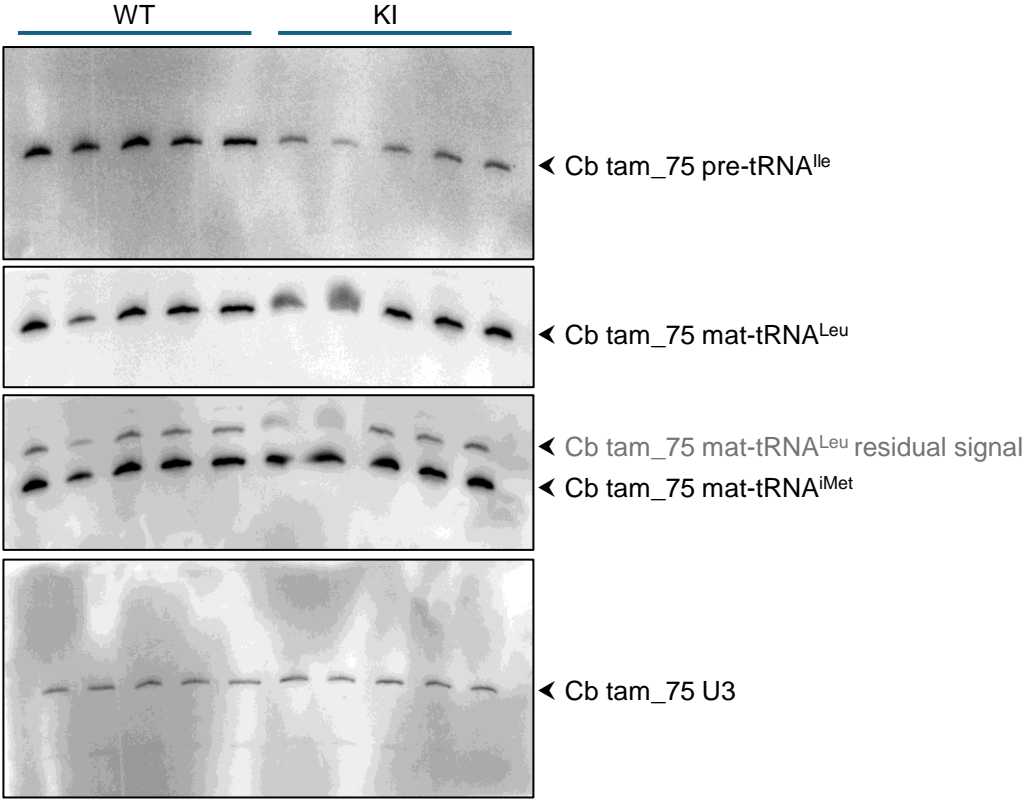

Heart

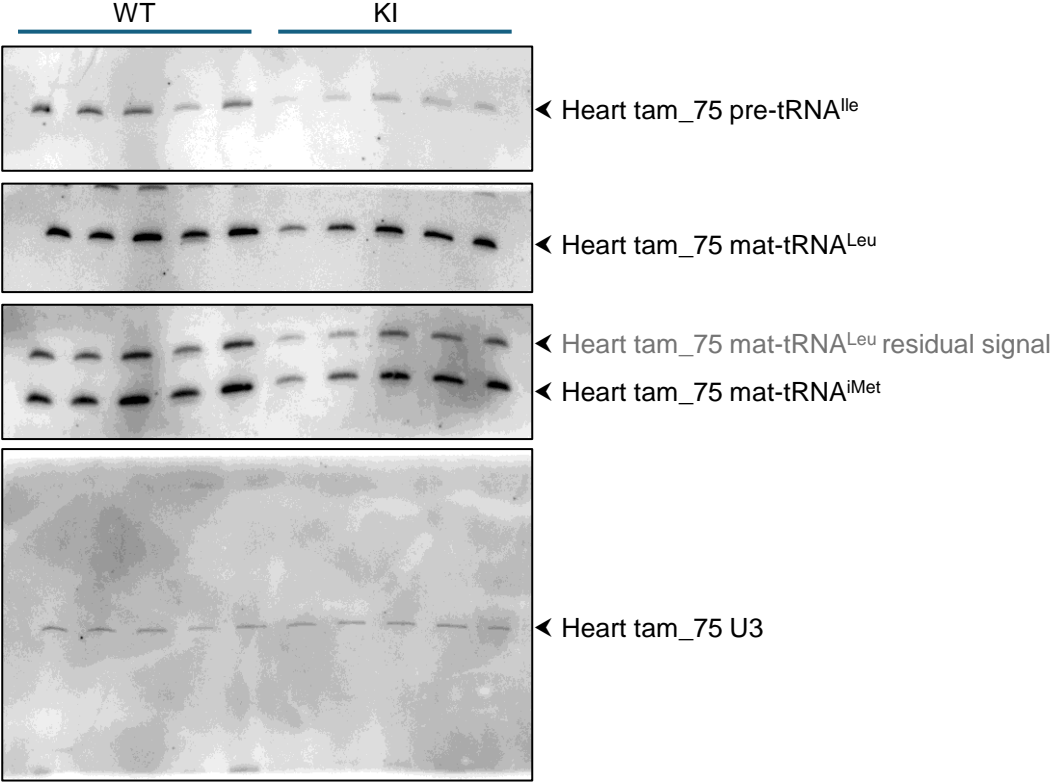

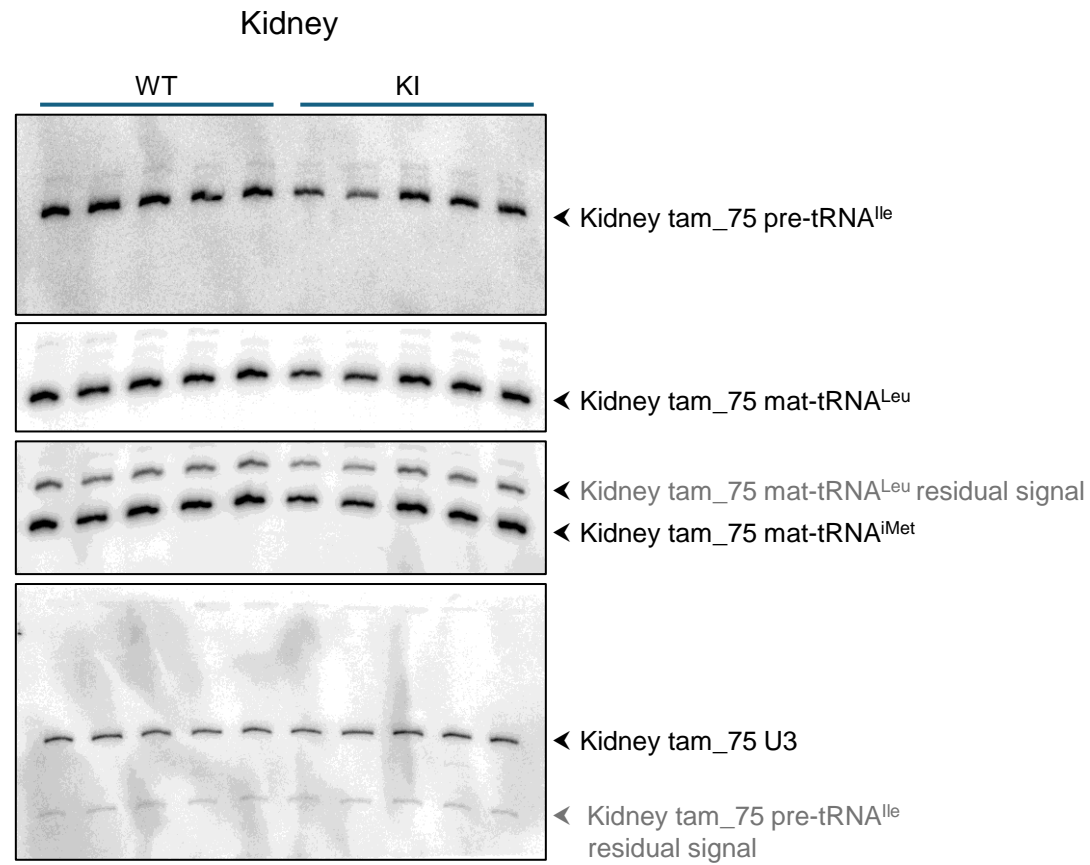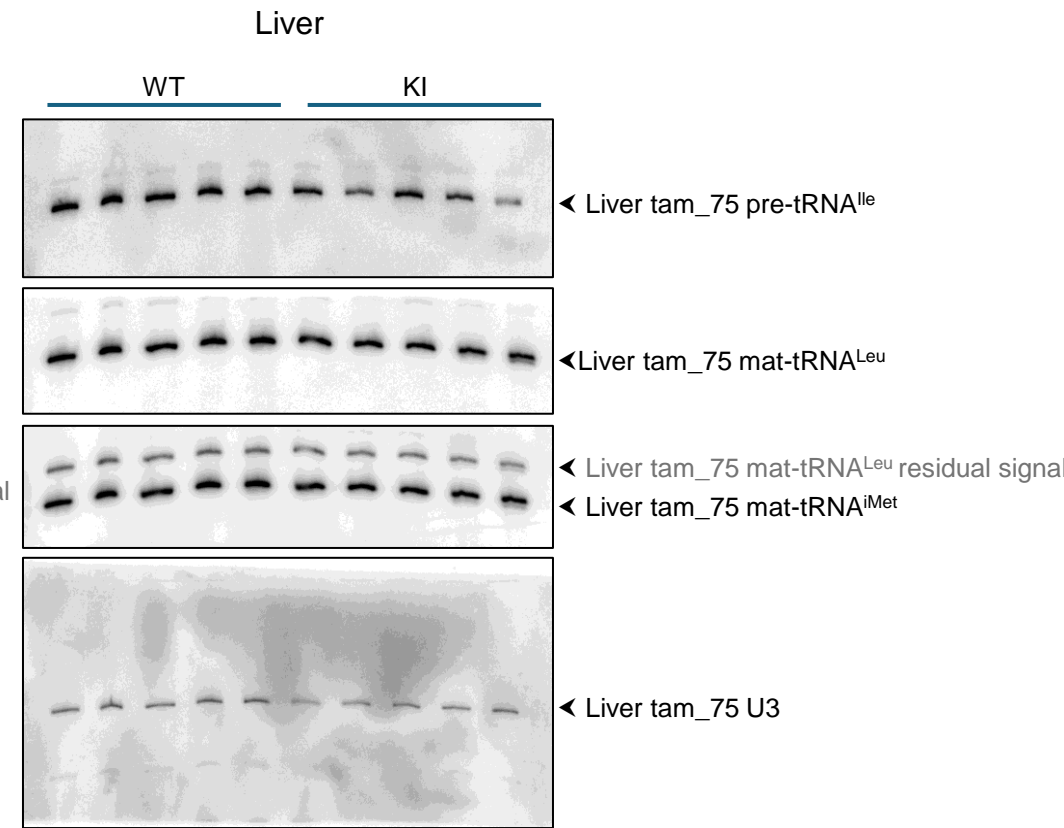

Supplement: Figure 3—figure supplement 2—source data 1. [file elife-95314-fig3-figsupp2-data1.pdf]

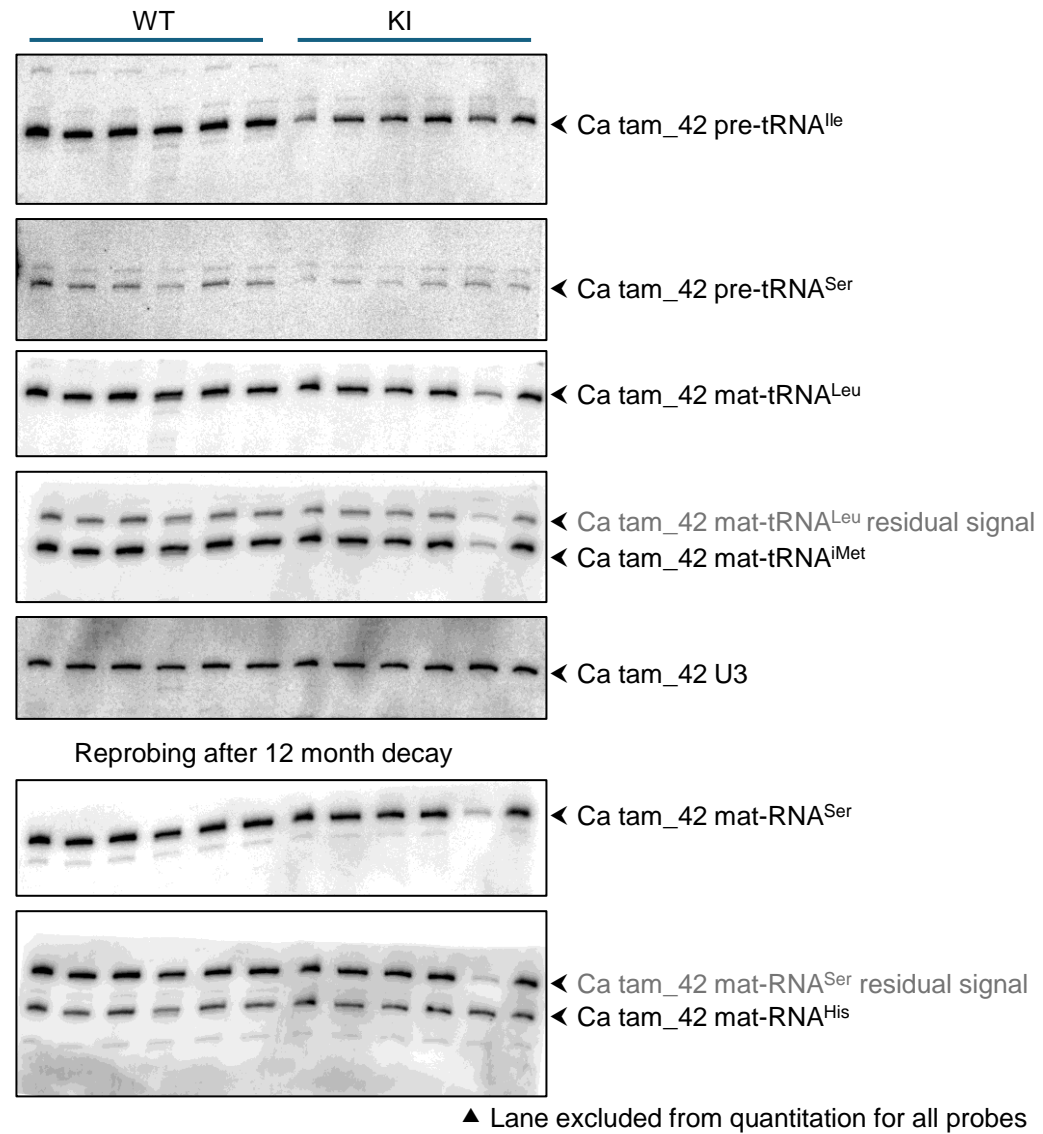

Supplement: Figure 5—source data 1. [file elife-95314-fig5-data1.pdf]
